# The art and science of a strategic grantmaker: the experience of the Public Health Agency of Canada’s Innovation Strategy

**DOI:** 10.17269/s41997-021-00512-9

**Published:** 2021-08-12

**Authors:** Shannon Bradley Dexter, Kelly Kavanagh Salmond, Leslie Payne, Marie C. Chia, Erica Di Ruggiero, Sarah Mahato

**Affiliations:** 1 Health Promotion and Chronic Disease Prevention Branch/Direction générale de la promotion de la santé et de la prévention des maladies chroniques, Public Health Agency of Canada/Agence de la santé publique du Canada, Ottawa, ON Canada; 2 Public Health Agency of Canada, 301-351 Abbott Street, Vancouver, BC V6B 0G6 Canada; 3 Dalla Lana School of Public Health, University of Toronto, Toronto, ON Canada

**Keywords:** Population health, Innovation, Population health intervention research, Grants and contributions, Health promotion, Public health, Mental health promotion, Social determinants of health, Healthy weights, Systems change, Santé de la population, innovation, recherche interventionnelle en matière de santé de la population, subventions et contributions, promotion de la santé, santé publique, promotion de la santé mentale, déterminants sociaux de la santé, poids santé, changement de systèmes

## Abstract

**Setting:**

The Public Health Agency of Canada’s Innovation Strategy (PHAC-IS) was established amid calls for diverse structural funding mechanisms that could support research agendas to inform policy making across multiple levels and jurisdictions. Influenced by a shifting emphasis towards a population health approach and growing interest in social innovation and systems change, the PHAC-IS was created as a national grantmaking program that funded the testing and delivery of promising population health interventions between 2009 and 2020.

**Intervention:**

During its decade-long tenure, the PHAC-IS supported the development of innovative, locally driven programs that emphasized health equity, encouraged iterative learning to respond reflexively to complex public health problems (the art), while at the same time promoting and integrating population health intervention research (the science) for improved health at the individual, community, and systems levels through four program components.

**Outcomes:**

PHAC-IS projects reached priority audiences in over 1700 communities. Over 1400 partnerships were established by community-led organizations across multiple sectors with more than $30 million of leveraged funds. By the final phase of funding, 90% of the projects and partnership networks had a sustained impact on policy and public health practice. By the end of the program, 82% of the projects were able to continue their intervention beyond PHAC-IS funding. Through a phased approach, projects were able to adapt, reflect, and build partnership networks to impact policy and practice while increasing reach and scale towards sustainability.

**Implications:**

Analysis and reflection throughout the course of this initiative showed that strong partnerships that contribute sufficient time to collaboration are critical to achieving meaningful outcomes. Building on evaluation cycles that strengthen project design can ensure both scale and sustainability of project achievements. Furthermore, a flexible, phased approach allows for iterative learning and adjustments across various phases to realize sustained population and systems change. The model and reflexive approach underlying the PHAC-IS has the potential to apply to a broad range of public programs.

## Résumé

**Contexte:**

La Stratégie d’innovation de l’Agence de la santé publique du Canada (SI de l’ASPC) a été établie sur fonds d’appels demandant divers mécanismes de financement structurel qui pourraient soutenir les programmes de recherche et orienter l’élaboration des politiques à plusieurs niveaux et dans de nombreuses administrations. Influencée par une réorientation vers une approche axée sur la santé de la population et un intérêt croissant pour l’innovation sociale et le changement de systèmes, la SI de l’ASPC a été créée en tant que programme national d’octroi de subventions finançant la mise à l’essai et l’exécution d’interventions prometteuses en matière de santé de la population de 2009 à 2020.

**Intervention:**

Au cours de ses dix ans de fonctionnement, la SI de l’ASPC a soutenu l’élaboration de programmes novateurs et locaux axés sur l’équité en santé et a encouragé l’apprentissage itératif afin de répondre instinctivement à des problèmes de santé publique complexes (l’art), tout en favorisant et en intégrant la recherche interventionnelle en santé de la population (la science) pour améliorer la santé au niveau des individus, des communautés et des systèmes au moyen de quatre éléments de programme.

**Résultats:**

Les projets de la SI de l’ASPC ont rejoint des publics prioritaires dans plus de 1 700 communautés. Plus de 1 400 partenariats ont été établis par des organisations communautaires dans plusieurs secteurs, et plus de 30 millions de dollars de fonds ont été mobilisés. À la phase finale du financement, 90 % des projets et réseaux de partenariat avaient une incidence durable sur les politiques et pratiques en matière de santé publique. À la fin du programme, 82 % des projets ont pu poursuivre leur intervention au-delà du financement de la SI de l’ASPC. Grâce à une approche progressive, les projets ont pu s’adapter, réfléchir et créer des réseaux de partenariat pour avoir une incidence sur les politiques et pratiques tout en augmentant leur portée et leur mise à l’échelle vers la durabilité.

**Implications:**

L’analyse et la réflexion tout au long de cette initiative ont démontré que de solides partenariats qui accordent suffisamment de temps à la collaboration sont essentiels à l’obtention de résultats significatifs. S’appuyer sur des cycles d’évaluation qui permettent de renforcer la conception des projets peut favoriser la mise à l’échelle et la durabilité des réalisations des projets. De plus, l’adoption d’une approche flexible et progressive permet un apprentissage itératif et des ajustements à différentes phases pour réaliser des changements durables au sein de la population et des systèmes. Le modèle et l’approche réflexive qui sous-tendent la SI de l’ASPC pourraient être appliqués à un large éventail de programmes publics.

## Introduction

Complex public health challenges require an approach that addresses social, economic, and environmental factors across multiple sectors and creates lasting impact at the population level. While investment in community-based projects has been a common approach within the field of health promotion and prevention, an opportunity exists to design programs to respond to the complexity of these challenges towards improved population health. Over a decade ago, the Public Health Agency of Canada designed an innovative funding model and approach that aimed to foster innovative and promising population health interventions that would have the potential to promote health at the individual, family, community, and systems levels.

The Public Health Agency of Canada-Innovation Strategy (PHAC-IS) was a national strategic program that supported the development of locally driven innovations between 2009 and 2020, while increasing the reach and impact of proven interventions towards long-term, sustained benefit. Drawing on theoretical underpinnings of social innovation, complexity thinking, and complex adaptive systems while incorporating a population health intervention research (PHIR) approach^1^, PHAC-IS investments^2^ reached priority audiences in over 1700 communities and impacted 2,070,920 individuals^3^.

The PHAC-IS was designed to support multi-sectoral partnerships for intervention delivery, community engagement, and knowledge exchange aimed at impacting policy and systems. Over 1400 partnerships were established by community-led organizations across multiple sectors, including municipal, regional, and provincial/territorial levels and the philanthropic and private sector. Using an intentional partnership approach, projects leveraged over $30 million of supplementary funds, in addition to in-kind resources through their partnership networks. By the final phase of funding^4^, 90% of the projects and partnership networks had a sustained impact on policy and public health practice. At the conclusion of the PHAC-IS in 2020, 82% of the projects were able to continue their intervention through funding from other sources or by partially or fully integrating into existing systems through scale-up. While all projects demonstrated an increase in protective factors and/or a decrease in risk factors among primary audiences at the individual level, the emphasis of this article is on the program model and approach to support promising interventions that aim to impact multiple levels across the individual, family, community, and systems for sustained population health promotion.

This article outlines the model, approach, and outcomes^5^ of the PHAC-IS as a way to contribute to dialogue and momentum for strategic grantmaking to promote population health. A combination of art and science guided the PHAC-IS model and approach across four program components, including a phased funding approach (grants and contributions), multi-level partnership development, knowledge development and exchange, and a focus on strengthening capacity and innovation (resources, methods, and tools). This article reflects on lessons learned, opportunities for improvement, and challenges identified along the way, punctuated with funding outcomes gained through rigorous performance monitoring and evaluation across three phases of funding. Learnings from the PHAC-IS model and reflexive approach, including emphasis on supporting partnerships, knowledge development and exchange, and scale-up aimed at impacting policy and systems to promote health, have the potential to apply to a broad range of public policies and programs.

## Background and setting

When the PHAC-IS was established in 2009, there was a paucity of information about how grantmaking models and initiatives could support social innovation and systems change. During that time, it was also recognized that integrating a population health approach to address the complexity of underlying determinants of health was extremely important and that a paradigm shift from a narrow focus on individual behavioural change to complex interventions that could effectively and equitably sustain impact at population and policy level was required (Butler-Jones, 2009; Hawe & Potvin, 2009; Hawe & Shiell, 2007; Nutbeam, 2001; Resnicow & Page, 2008). The literature also pointed to a need for diverse structural funding mechanisms that could support research agendas to mobilize knowledge gained from these interventions, including the scale-up of pilot projects into long-term funding programs and knowledge sharing across jurisdictions (CIHR-IPPH, 2010; Bégin, 2009).

In response, the PHAC-IS focused on social innovation and systems change within a PHIR approach. Social innovation has been described by Broadhead (2010) as “a complex process of introducing products, processes or programs that profoundly change the basic routines, resources and authority flows or beliefs of the social system in which they arise.” A nested theory within the PHAC-IS model was that of complex adaptive systems change and the notion that difficult public health challenges require a robust and multifaceted response that interacts with and promotes health at the individual, family, and community levels (Resnicow & Page, 2008). The PHAC-IS was also informed by a concept of strategic grantmaking that had gained momentum in private and philanthropic sectors since the 1990s, in part because of its ability to jump-start innovation and its emphasis on broad impact and long-term goals (Orfield et al., 2015). This concept emphasized that greater intentionality and strategic processes contribute to a strong theory of change (Easterling & Metz, 2016).

Additionally, in 2006, an Independent Blue Ribbon Panel on Grants and Contributions called for greater accountability across government departments at the federal level while improving both efficiency and access. The Blue Ribbon Panel recommended increased focus on the design of strategic programs as well as innovation and the importance of identifying intended results at the outset (Treasury Board Secretariat of Canada, 2006). In response, PHAC initiated a process to design a model that would address barriers encountered by funders and grant recipients such as limited resources and lack of capacity to generate evidence as well as lessons learned from successful interventions (PHAC, 2015). A priority-setting exercise with federal departments, provincial and territorial ministries, and key non-governmental organizations and stakeholders identified the need for evidence-based population health interventions within two priority streams, *Equipping Canadians: Mental Health Throughout Life* (2009–2018) and *Achieving Healthier Weights in Canada’s Communities* (2011–2020).

By design, the PHAC-IS model enhanced a culture of innovation and learning within population health interventions to reduce health inequities and effectively address priority public health problems and their underlying factors. In order to remain responsive, a combination of art and science guided the PHAC-IS model. The “art” refers to a reflexive, partnership-driven model with a focus on continual improvement to foster innovation and adapt to complex population health challenges operating alongside the “science” of PHIR and the four components of the model: phased funding approach (grants and contributions), partnership development, knowledge development and exchange, and strengthening capacity and innovation (resources, methods, and tools). Two overarching objectives guided the iterative aspects of the PHAC-IS: supporting the development, adaptation, implementation, and evaluation of promising innovative population health interventions to address priority challenges and their underlying factors, and supporting knowledge development and exchange based on the systematic collection of data across three phases (Table 1).

**Table 1:** PHAC-IS across three phases of funding (up to nine years)

Program objectives		
• To support the development, adaptation, implementation, and evaluation of promising national and international innovative population health interventions and initiatives to address priority issues and their underlying factors in various settings and populations across Canada. • To support knowledge development and exchange based on the systematic collection of the results and outcomes of these interventions and initiatives and promote their use across Canada.		
Funding phases and timelines		
Phase 1 12–18 months	Phase 2 4 years	Phase 3 3 years
To support the initial design, development, and testing of interventions	To support the implementation, delivery, and evaluation of population health interventions in multiple sites	To scale up effective population health interventions
Eligible funding (per project)		
$150,000 to $250,000/year	$300,000 to $750,000/year	Up to $500,000/year
Number of projects funded		
MHP: 15	MHP: 9	MHP: 4
AHW: 37	AHW: 11	AHW: 7
Reach of funded interventions among individuals or those who face specific risk conditions or risk factors		
MHP: 3358	MHP: 281,742	MHP: 182,818
AHW: 59,081	AHW: 21,943	AHW: 87,846
# and % of projects demonstrating change in health outcomes, protective factors, and/or risk behaviours		
N/A*	9 (100%) of MHP projects	4 (100%) of MHP projects
8 (22%) AHW projects	7 (64%) of AHW projects †	7 (100%) of AHW projects

*MHP*, Equipping Canadians: Mental Health Throughout Life funding stream; *AHW*, Achieving Healthier Weights in Canada’s Communities funding stream; *N/A*, not applicable

*Given that this was an intermediate outcome with a 4–6-year time frame and project performance evaluation and reporting forms completed at the time captured only information from the first year of implementation, many projects could not yet respond to this question. Either projects had not attempted to directly improve health practices, skills, or outcomes at this stage or this had not yet been sufficiently measured to conclusively confirm a change had occurred

^†^This figure represents behaviour change only for phase 2 projects funded through the Achieving Healthier Weights in Canada’s Communities stream; 55% (6/11) projects reported a change in protective factors among participants. Additionally, 45% (5/11) of projects reported improved well-being among participants

## Review and evaluation methods

The PHAC-IS operated for approximately 11 years using an analytical and structured reflection process that was integrated into its delivery to foster a culture of innovation and learning (Cook & Bradley Dexter, 2021). The findings in this article are drawn from this process and include highlights related to the four components of the program. To capture these highlights, an analysis of project and program sources was completed, including independent consultant reports at the project and program level, project knowledge products, PHAC-IS reports, and PHAC corporate evaluations.

In addition, rigorous performance monitoring captured the outcomes of PHAC-IS funding across the four components. Performance measurement tools and processes developed for evaluation reviews^6^ of funded projects (Boileau-Falardeau et al., 2021) as well as program-level performance measurement informed the analysis. Although qualitative examples of projects are provided to illustrate components of the funding model, this article does not include an overview of specific outcomes from the funded projects.

## Intervention and outcomes

**Fig. 1:**
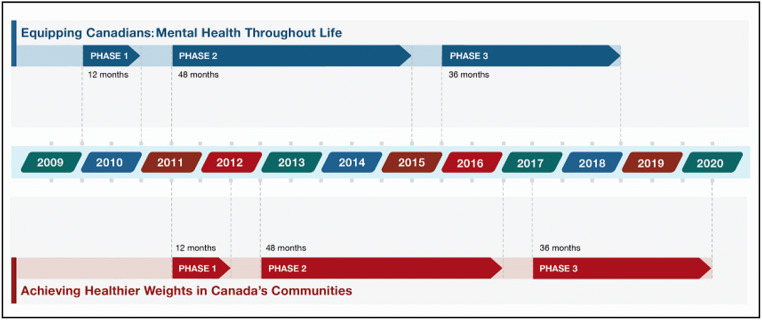
PHAC-IS funding phases: 2009–2020

As noted, the PHAC-IS emphasized four components: phased funding approach (grants and contributions), partnership development, knowledge development and exchange, and strengthening capacity and innovation (resources, methods, and tools). These components were informed by a PHIR approach that acknowledged intersectoral partnership development, multiplicity, and intervention implementation quality as a subset of PHIR and as key objective, knowledge exchange (Riley et al., 2015). Each component was developed, implemented, monitored, and evaluated to advance the PHAC-IS objectives in an iterative way through complementary work at the project, project cohort, and program levels. To provide a robust picture of the PHAC-IS, outcomes are framed along the four core components (Fig. 1 and Table 1). The components were also influenced by cross-cutting principles, including social innovation, multi-level and multi-sectoral action, cultural safety, health equity, and evidence-based decision-making.

1. A phased approach to social innovation and systems change (grants and contributions)

The PHAC-IS approach acknowledged that high-quality innovative population health interventions must be developed, tested/piloted, and assessed during a multi-year development cycle. Originally conceived with two phases, it was observed that a third phase would provide effective interventions with the opportunity to move towards scale-up to increase reach and impact systems change (Bradley Dexter et al., 2021). Phase 1 supported the initial design, development, and testing of an intervention; phase 2 focused on full implementation to additional sites alongside rigorous evaluation; and finally, phase 3 provided funding for scale-up for extended reach and sustained impact^7^. A three-phased approach to long-term funding Fig. 1) allowed for and supported the lengthy processes involved in social innovation and systems change that traditional shorter models of funding were unable to address.

In line with the Treasury Board’s Blue Ribbon Panel recommendations on grants and contributions, the PHAC-IS’s phased approach (Text Box 1) was competitive and provided a way for projects to receive funding for up to 9 years, though application to subsequent phases was voluntary and at the discretion of the project (Bradley Dexter et al., 2021). This approach facilitated a stable funding framework to develop lasting partnerships and to implement, test, and adapt work while performing high-quality research that focused on scale-up and longer-term impact. Exit interviews with funded projects revealed that the phased application process focussed the host organization to intentionally design their partnership network and intervention expansion with consideration for scale-up and systems change.

**Text Box 1:** The PHAC-IS phased approach to funding

• **Phase 1: Initiation and development** focused on the early design, development, testing and delivery of population health interventions over an initial period of 12 to 18 months (budgets from $150,000 to $250,000 per year). Phase 1 supported the development of vested partnerships and networks among public, non-profit, community, academic and private sectors. **• Phase 2: Delivery and evaluation** supported full implementation, adaptation and evaluation of comprehensive population health interventions across multiple populations, communities (in at least three different jurisdictions) and settings over a period of up to four years (budgets from $300,000 to $750,000 per year). Phase 2 created and distributed evidence on community and cultural context compatibility, implementation readiness and the measurable impact of population health promotion interventions. **• Phase 3: Scale-up successes** supported efforts to expand the reach and impact of successfully evaluated population health interventions (budgets up to $500,000 annually). This funding was available for a three-year period to build capacity and create opportunities to scale up the reach to additional populations and policy impact of proven interventions to sustain the measurable impact.	

In phase 1, applicants were assessed based on requirements that reflected a population health approach combined with early predictors of their capacity to develop, deliver, evaluate, and build partnerships. By the end of phase 1, funded interventions had reached over 60,000 individuals affected by specific risk conditions or factors, with some already demonstrating changes in health outcomes and risk/protective factors within an 18-month time frame (Table 1). During phase 2, projects developed evaluation plans and expanded, reaching over 1000 communities across all provinces and territories. In phase 3, applicants were assessed based on the capacity of their intervention to scale up. Assessment criteria were derived from PHAC-IS research and analysis to identify predictors of success in scale-up for population health interventions. These domains included: intervention evidence and evaluation, reach and scale, organizational capacity, partnership development, system readiness, community context, cost factors, and knowledge development and exchange (Bradley Dexter et al., 2021).

As noted, by the completion of phase 3, 82% of projects were able to sustain all or part of their intervention activities after PHAC funding had completed. In addition, there were 44 instances across 90% of the projects in phase 3 of policy and practice change, spanning a range of local, regional, provincial, and territorial jurisdictions. Through the phased approach, projects were able to adapt, reflect, and build additional partnership networks to impact policy and practice while increasing scale towards sustainability (Bradley Dexter et al., 2021).

(2) Partnership development

A strong partnership approach supported funding to build capacity for delivery, knowledge mobilization, and policy development in several domains. While many funders support and monitor partnership development, the PHAC-IS recognized this as a foundational component of scale-up and eventual systems change. Partnership development and collaboration with practitioners, researchers, policy makers, and community organizations was embedded in project planning and collaborations spanned local governments, municipalities, regional health authorities, and provincial/territorial governments, as well as private sector organizations. By phase 2, almost 40% of the over 1400 partnerships developed among the 20 funded projects had been sustained for 3 or more years. As the funded projects scaled up in phase 3, the average number of partnerships per project remained steady above 50% and over half (58%) were maintained for 3 or more years. This time, investment to build and nurture partnerships was an essential feature for projects that were able to work well within the communities that they served, scale with purpose, and ultimately change systems. An excerpt from a recent PHAC-IS evaluation report highlights this in a project spotlight from two phase 2 funded projects (Text Box 2).

**Text Box 2:** Project spotlight: Partnerships

***Equipping Canadians: Mental Health Throughout Life*** The *Towards Flourishing: Mental Health Promotion for Families* project, led by the University of Manitoba, formed partnerships with a wide variety of stakeholders, including provincial ministries of health, regional health authorities, the Assembly of Manitoba Chiefs and the First Nations Advisory Group. Partners brought a wide range of experience, knowledge and skills to the project and contributed to improving and promoting the mental well-being of parents and their families. These strong partnerships ultimately led to this project being delivered through the Province of Manitoba to support the *Towards Flourishing* strategy across the province.	
***Achieving Healthier Weights in Canada's Communities*** *Active Neighbourhoods Canada/Réseau Quartiers verts* led by the Montreal Urban Ecology Centre/Centre d’écologie urbaine de Montréal had 60 stakeholders by the end of Phase 3 in health, transportation, planning, environment, social services and research who were engaged to conduct community needs assessments and create social and physical environments that supported active transportation. (Office of Evaluation, Health Canada and the Public Health Agency of Canada, 2019)	

An analysis of “vested partnerships” within the PHAC-IS highlights characteristics of a robust partnership approach towards systems change. Vested partnerships have diverse partners, a clear public sectoral agenda, demonstrated collaborative value, often a pooling of both human and financial assets, and a commitment to alignment and dialogue, along with a collective approach for collaborative systems change (Lee & Kavanagh Salmond, 2021). Although partnership development was anticipated in phase 2, early learnings indicated that more time was needed to develop meaningful connections at the project level. Significant time and funding were also required to conduct outreach, meet with partners, and establish ongoing, meaningful, and sustained relationships.

This shifting emphasis on capturing the *vestedness* of partnerships also has implications for performance monitoring efforts which should aim to capture the movement of the collaborative effort, including agenda setting, rather than the number, or reach, of partners in a network. In light of this, the PHAC-IS offers the duration of partnerships as a limited proxy for vestedness, as well as an introspective commentary around the challenges of collecting and contextualizing information for performance and evaluation measurement purposes (Boileau-Falardeau et al., 2021).

(3) Knowledge development and exchange

Knowledge development and exchange (KDE) was directed at developing strategies to share evidence-based knowledge products, activities, and lessons learned to impact future public health practice, program, and policy development. The combined emphasis on partnership and knowledge exchange resulted in over 400 examples from phase 2 projects of how knowledge generated by their intervention work was applied, and over 78% of these examples directly informed or led to the implementation or adaptation of a practice, policy, or program.

In phase 3, greater emphasis was placed on the effective uptake of knowledge among internal and external partners and other stakeholders (potential knowledge users who were external to the project) rather than the quantity of knowledge products. Projects continued to create and deliver thousands of knowledge products and activities, and a greater number of these were designed to influence policy, practice, and programming. Projects cited over 200 examples of how the knowledge generated by their intervention work influenced policy, practice, and/or programs. As outlined in Text Box 3, phase 3 projects were able to work with their partners to influence policy change at regional, provincial, and territorial levels and adapt public health programming. The PHAC-IS influenced a range of sectors, including: municipal transportation plans to encourage physical activity, regional plans to support local food security, and school districts adopting social and emotional learning curricula. The creation of KDE plans was stipulated as part of the work plan, and this led to dedicated time and resources allocated to the KDE process at the project level. The long-term funding of up to 9 years combined with partnership development and KDE allowed projects to redesign their approach through each phase and continue to maximize the uptake of knowledge/evidence into practice and policy decisions that effected change within complex public health issues.

**Text Box 3:** Project spotlight: Knowledge development and exchange

***Equipping Canadians: Mental Health Throughout Life*** Knowledge development and exchange initiatives of the *Fourth R: Healthy Relationships* project led by the Centre for Addiction and Mental Health (CAMH) and Western University supported the inclusion of a *Safe Schools* graduate course for teacher candidates at the University of Calgary and the University of Lethbridge. This project also informed the development of anti-bullying policies and legislation in the Northwest Territories. ***Achieving Healthier Weights in Canada's Communities*** The *Expanding the Impact and Reach of Community Food Centre Model* project led by Community Food Centres Canada provided access to knowledge, training and resources to community food security organizations across the country through its *Good Food Organizations* program. The Good Food Organizations program is helping community food security organizations change their programmatic or organizational policies. An annual assessment of the program demonstrated the following examples of internal policy change in member organizations: • 49% of the Good Food Organizations created or deepened a healthy food policy that guides purchases and menu choices; • 93% broadened their food programming to serve multiple objectives in the area of food access, food skills, and community engagement; and • 73% increased material supports to enable low-income and marginalized people to participate in their programs. The *Healthy Start / Départ Santé Project (HSDS): A Multi-Level Intervention to Increase Physical Activity and Healthy Eating Among Young Children (3-5) Attending Early Learning Programs* project led by Réseau Santé en français de la Saskatchewan (RSFS) implemented knowledge development and exchange (KDE) teams to deliver training and information sessions to early childhood educators and administrators of child care centres. Through the promotion of best practices, individual champions and booster training sessions, several opportunities for training and support were made available. As an early indication of knowledge uptake, after training, 94% of centres were using the physical activity materials and 95% were using the nutrition resources at least two times per month. *Our Food NL* project led by Food First Newfoundland implemented an innovative Community-Led Food Assessment (CLFA) model. A CLFA helps a community to identify gaps and strengths to access healthy and culturally appropriate food, so they may initiate effective solutions to local food security challenges. This effort brought together a broad range of partners at the community, regional, provincial, and national levels to address serious food security challenges facing communities. The CLFA model has had a lasting impact on policy and programming in several communities in Newfoundland and Labrador. For example, the CLFA led to concrete changes in the availability and diversity of fresh produce offered in community stores. In Rencontre East (accessible only by ferry), a local storeowner created a successful points system where customers accumulate points by purchasing fresh fruits and vegetables, and at the end of the month, the customer with the most points receives a prize. In response to the CLFA findings, this private business began offering single portions of fruits and vegetables to respond to feedback from seniors (Our Food NL, 2018).	

Measuring the use of the knowledge products and activities by other stakeholders was challenging for projects; however, by phase 3, all projects were able to identify how knowledge generated through their work impacted policy and practice^8^. Despite the relative successes of KDE by projects, the ability to impact systems change requires a responsive and adaptive audience. KDE also requires expertise, time, and resources to build the links between the research and evaluation agenda of an intervention to the knowledge needs of a program designer or policy maker. For future iterations of this model, an interactive, external platform for KDE is expected to provide capacity building to the projects to support the design of evaluation plans with an integral KDE plan, alongside facilitating the uptake of knowledge by developing a community of practitioners and policy makers as knowledge brokers and users.

(4) Strengthening capacity and innovation (resources, methods, and tools)

The PHAC-IS supported projects by creating or supplementing many resources, methods, and tools to strengthen capacity and access to information on a variety of topics, including implementation science, knowledge mobilization, collective impact, policy change, and scale-up. These were often delivered in partnership and made available to wider audiences (e.g., webinars with the National Collaborating Centre for Methods and Tools) or through formalized connections between the PHAC-IS and project partners (e.g., PHAC-IS technical presentations to project advisory boards). In addition, the PHAC-IS team organized annual multi-day workshops for funded projects to meet with experts in various fields and exchange structured lessons learned on topics related to implementation science, partnership development, scale-up, and evaluation. These themes were identified by projects to respond to specific capacity-building needs, and by phase 3, projects led the development, facilitation, and content creation of the annual workshops.

A gap in capacity for evaluation was identified during phase 1 and the majority of projects partnered with a university or researcher to provide the rigour to evaluate their intervention and collect sufficient data for PHIR. In addition, PHAC-IS supported the development of several evaluation resources and engaged an evaluation expert to support projects with return on investment analysis, the evaluation of policy influence, and one-on-one support for tailored evaluation plans. Projects were very diverse in context, population served, and intervention approach; as a result, a tool kit or generic resource for PHIR often was not as effective as one-on-one approaches. Within future iterations of the funding model, tailored capacity building and supports will be built into the funding model starting in phase 1 and further integrated and supported through an external knowledge development and exchange hub. It was also difficult to capture the impact of capacity-building tools, and future iterations of the funding model will link this component to the KDE and partnership work towards systems change.

## Implications

The PHAC-IS model has informed the design of other funding initiatives provided by the Canadian Institutes of Health Research-Institute of Population and Public Health (CIHR, 2016a; CIHR-IPPH, 2010) and the *Pathways to Health Equity for Aboriginal Peoples Initiative* (CIHR, 2016b) as well as the subsequent PHAC funding programs *Supporting the Health of Survivors of Family Violence Program*, the *Mental Health of Black Canadians Fund*, and the *Dementia Community Investment Fund* (Office of Evaluation, Health Canada and the Public Health Agency of Canada, 2019).

There are a number of important lessons learned during the PHAC-IS journey that are useful to those administering and managing strategic funding programs for health promotion at the individual and system levels. First, strong, vested partners were key to the delivery, implementation, scale-up, and sustainability of projects in both funding streams. These partnerships led to a valuable two-way exchange of knowledge that improved practice toward systems change. Analyses of vested partnerships offered the need to capture the collective movement and shared agenda (Lee & Kavanagh Salmond, 2021). Exit interviews from phase 3 projects also highlighted the advantage of time and space to build out partnerships as a means to focus efforts upstream towards systems change. Therefore, flexibility and support for partnership and, ultimately, relationship development and shared agenda-setting activities have the potential to create scale-up and sustainability in programming.

Second, incorporating evaluation expertise in each funded project ensured that interventions were tested to better understand the impact of their activities, which added to the evidence base on effective approaches. The PHAC-IS provided support to enhance understanding of what was meant by “scale-up” (Bradley Dexter et al., 2021) and how to move towards policy and systems change. As a result, projects were able to take the necessary steps to expand their interventions and enhance sustainability.

Third, the PHAC-IS phased approach built in flexibility that allowed the program and projects to identify and learn from what worked and what did not at the end of each phase. This resulted in course corrections that would improve overall success.

Finally, the length and potential for prolonged funding across the three phases was seen as especially vital for this type of program, since projects were trying to create long-lasting system-level changes that can only be achieved through years of committed investment (Office of Evaluation, Health Canada and the Public Health Agency of Canada, 2019).

Challenges were also revealed throughout this iterative process. While the PHAC-IS supported projects that sought to address health inequities, it was sometimes difficult to capture impact in a meaningful way, in part because of the complexity and diversity in the context at the project level. Future iterations of this funding program will incorporate opportunities and methods at the start of the funding cycles to capture information on how projects approach and impact health inequities.

In addition, despite the relative success of integrating KDE at the program level for continued learning, the depth and range of work presented challenges. Subsequent iterations of the PHAC-IS model are exploring additional resources for an external organization that will support projects to develop rigorous evaluation plans, and facilitate collaborative partnership experiences, shared learnings and knowledge exchange among and outside of funded projects to advance a more enabling environment for social innovation towards systems change.

## Conclusion

Strategic and responsive grantmaking programs have the potential to support innovative and locally grown solutions to tackle complex public health issues. The PHAC-IS model supported creative local action to promote positive and sustainable change in systems and jurisdictions in Canada’s communities and beyond. The PHAC-IS model accomplished this by using a phased approach with an emphasis on partnership development, knowledge development and exchange, and strengthened capacity and innovation. Across the four core components of the PHAC-IS model, evidence from innovative local solutions to complex challenges influenced and informed policies and practice across sectors and throughout jurisdictions. PHAC-IS reflections and funding outcomes are offered here to encourage more dialogue about health promotion grantmaking and programming among multiple levels of government and community organizations that seek to reduce risk factors, promote protective factors, and address their underlying determinants of health through population health interventions.

Achievements at the program and project levels through longer-term, phased funding supported design, delivery, and eventual scale-up, as well as strong and vested partnerships that were key to knowledge exchange that improved practice and supported health-promoting policies. The PHAC-IS combined the art of innovation and continuous improvement with the science of sound public health implementation as an intentional basis to guide the strategic design of a funding model to tackle the complex public health challenges in Canada’s communities.

**L’art et la science de la subvention stratégique: l’expérience de la Stratégie d’innovation de l’Agence de la santé publique du Canada**

## Introduction

Les défis complexes de santé publique exigent une approche tenant compte des facteurs sociaux, économiques et environnementaux dans de multiples secteurs et générant une incidence durable au niveau de la population. Bien que l’investissement dans des projets communautaires soit une approche courante dans le domaine de la promotion de la santé et de la prévention, il est possible de concevoir des programmes qui répondent à la complexité de ces défis en vue d’améliorer la santé de la population. Il y a plus de dix ans, l’Agence de la santé publique du Canada a conçu une approche et un modèle de financement novateurs visant à favoriser les interventions novatrices et prometteuses en matière de santé de la population qui pourraient promouvoir la santé au niveau des individus, des familles, des communautés et des systèmes.

La Stratégie d’innovation de l’Agence de la santé publique du Canada (SI de l’ASPC) était un programme stratégique national qui a soutenu le développement d’innovations locales de 2009 à 2020, tout en augmentant la portée et l’incidence des interventions éprouvées pour produire des avantages durables à long terme. S’appuyant sur les fondements théoriques de l’innovation sociale, de la réflexion sur la complexité et des systèmes adaptatifs complexes tout en utilisant une approche de recherche interventionnelle dans le domaine de la santé de la population (RIDSP)^9^, les investissements^10^ de la SI de l’ASPC ont rejoint des publics prioritaires dans plus de 1 700 communautés et ont touché 2 070 920 individus^11^.

La SI de l’ASPC a été conçue pour soutenir les partenariats multisectoriels à des fins de prestation d’interventions, de mobilisation communautaire et d’échange de connaissances visant à influencer les politiques et les systèmes. Plus de 1 400 partenariats ont été établis par des organisations communautaires dans de multiples secteurs, y compris aux niveaux municipal, régional et provincial ou territorial et les secteurs philanthropique et privé. En utilisant une approche de partenariat intentionnel, les projets ont mobilisé plus de 30 millions de dollars en fonds supplémentaires, en plus des ressources en nature obtenues par le biais de leurs réseaux de partenariats. À la phase finale du financement^12^, 90 % des projets et réseaux de partenariats avaient une incidence durable sur les politiques et pratiques en matière de santé publique. À la fin de la SI de l’ASPC en 2020, 82 % des projets ont pu poursuivre leur intervention grâce au financement provenant d’autres sources ou en l’intégrant partiellement ou entièrement à des systèmes existants grâce à une mise à l’échelle. Bien que tous les projets aient démontré une augmentation des facteurs de protection ou une diminution des facteurs de risque chez les principaux publics au niveau individuel, le présent article porte sur le modèle de programme et l’approche utilisés pour soutenir les interventions prometteuses visant à avoir une incidence à plusieurs niveaux chez les individus, les familles, les communautéss et les systèmes pour promouvoir de façon durable la santé de la population.

Le présent article décrit le modèle, l’approche et les résultats^13^ de la SI de l’ASPC comme moyen de contribuer au dialogue et à donner l’élan à l’octroi de subventions stratégiques afin de promouvoir la santé de la population. Le modèle et l’approche de la SI de l’ASPC ont été orientés au moyen d’une combinaison d’art et de science dans les quatre éléments du programme, comprenant une approche progressive de financement (subventions et contributions); l’établissement de partenariats à plusieurs niveaux; le développement et l’échange de connaissances; et un accent sur le renforcement des capacités et l’innovation (ressources, méthodes et outils). Le présent article se penche sur les leçons tirées, les possibilités d’amélioration et les défis décelés en cours de route, ainsi que sur les résultats du financement obtenus grâce à un suivi rigoureux et une évaluation du rendement au cours des trois phases de financement. Les leçons tirées du modèle et de l’approche réflexive de la SI de l’ASPC, y compris l’accent mis sur le soutien des partenariats, le développement et l’échange des connaissances, et la mise à l’échelle afin d’avoir une influence sur les politiques et les systèmes en matière de promotion de la santé, pourraient être appliquées à un large éventail de politiques et de programmes publics.

## Contexte et milieu

Lors de la création de la SI de l’ASPC en 2009, il y avait peu de données sur la façon dont les modèles et les initiatives d’octroi de subventions pouvaient soutenir l’innovation sociale et le changement de systèmes. Il avait alors également été reconnu qu’il était extrêmement important d’intégrer une approche axée sur la santé de la population pour aborder la complexité des déterminants sous-jacents de la santé et nécessaire de passer d’un accent étroit sur les changements de comportements individuels à des interventions complexes qui pourraient efficacement et équitablement maintenir l’incidence au niveau de la population et des politiques (Butler-Jones, 2009; Hawe & Potvin, 2009; Hawe & Shiell, 2007; Nutbeam, 2001; Resnicow & Page, 2008). La documentation a aussi souligné la nécessité de fournir divers mécanismes de financement structurel pouvant soutenir les programmes de recherche afin de mobiliser les connaissances tirées de ces interventions, y compris pour la mise à l’échelle de projets pilotes en programmes de financement à long terme et le partage de connaissances entre les administrations (CIHR-IPPH, 2010; Bégin, 2009).

La SI de l’ASPC s’est donc concentrée sur l’innovation sociale et le changement de systèmes dans le cadre d’une approche de RIDSP. L’innovation sociale a été décrite par Broadhead (2010) comme « un processus complexe consistant à présenter des produits, des processus ou des programmes qui modifient profondément les habitudes de base, la répartition des ressources et des pouvoirs ou les croyances du système social où ils se manifestent. » Une théorie imbriquée dans le modèle de la SI de l’ASPC était celle du changement de systèmes adaptatifs complexes et la notion selon laquelle les défis difficiles en matière de santé publique exigent une réponse robuste et multiforme qui interagit avec la santé et la favorise au niveaux individuel, familial et communautaire (Resnicow & Page, 2008). La SI de l’ASPC a également été orientée par un concept d’octroi de subventions stratégiques ayant pris de l’ampleur au sein des secteurs privé et philanthropique depuis les années 1990, en partie en raison de sa capacité à relancer l’innovation et de l’accent mis sur la large influence et les objectifs à long terme (Orfield et al., 2015). Ce concept souligne qu’une plus grande intentionnalité et des processus stratégiques contribuent à une solide théorie du changement (Easterling & Metz, 2016).

De plus, en 2006, un groupe d’experts indépendant sur les subventions et les contributions a appelé à une plus grande responsabilisation des ministères fédéraux et à l’amélioration de l’efficacité et de l’accès. Le groupe d’experts a recommandé de mettre davantage l’accent sur la conception de programmes stratégiques ainsi que sur l’innovation et l’importance d’identifier les résultats attendus dès le départ (Treasury Board Secretariat of Canada, 2006). L’ASPC a donc lancé un processus de conception d’un modèle qui éliminerait les obstacles rencontrés par les bailleurs de fonds et les bénéficiaires de subventions, comme les ressources limitées et le manque de capacité à produire des données probantes ainsi que des leçons tirées des interventions fructueuses (PHAC, 2015). Un exercice d’établissement des priorités avec les ministères fédéraux, provinciaux et territoriaux et des organisations non gouvernementales et intervenants clés a établi la nécessité d’appuyer des interventions en matière de santé de la population fondées sur des données probantes dans deux secteurs prioritaires : *Outiller les Canadiens – La santé mentale pour la vie* (2009 à 2018) et *Atteinte du poids santé dans les collectivités du Canada* (2011 à 2020).

De par sa conception, le modèle de la SI de l’ASPC a favorisé une culture d’innovation et d’apprentissage dans les interventions en matière de santé de la population afin de réduire les inégalités en santé et de s’attaquer efficacement à des problèmes de santé publique prioritaires et leurs facteurs sous-jacents. Afin de favoriser l’adaptabilité, le modèle de la SI de l’ASPC a été orienté au moyen d’une combinaison d’art et de science. « L’art » fait référence à un modèle axé sur la réflexion, les partenariats, ainsi que sur l’amélioration continue afin de favoriser l’innovation et de s’adapter aux défis complexes en matière de la santé de la population, et s’applique parallèlement à « la science » de la RIDSP et aux quatre éléments du modèle : approche progressive de financement (subventions et contributions); établissement de partenariats; développement et échange des connaissances; et renforcement des capacités et de l’innovation (ressources, méthodes et outils). Deux objectifs généraux ont guidé les aspects itératifs de la SI de l’ASPC : appuyer l’élaboration, l’adaptation, la mise en œuvre et l’évaluation d’interventions novatrices et prometteuses en matière de santé de la population qui visent à régler des problèmes prioritaires et influer sur leurs facteurs sous-jacents; et soutenir le développement et l’échange de connaissances en s’appuyant sur la collecte systématique des données au cours des trois phases (Tableau 1).

**Tableau 1:** Les trois phases de financement de la SI de l’ASPC (jusqu’à neuf ans)

Objectifs du programme		
• Appuyer l’élaboration, l’adaptation, la mise en œuvre et l’évaluation d’interventions et d’initiatives prometteuses et novatrices, aussi bien nationales qu’internationales, qui visent à améliorer la santé de la population en vue de régler des problèmes clés et d’influer sur leurs facteurs sous-jacents dans divers milieux et dans diverses populations du Canada. • Soutenir le développement et l’échange des connaissances en s’appuyant sur la collecte systématique des résultats des interventions et des initiatives et la promotion de leur utilisation partout au Canada.		
Phases et calendriers du financement		
Phase 1 12 à 18 mois	Phase 2 4 ans	Phase 3 3 ans
Soutenir la conception initiale, l’élaboration et la mise à l’essai des interventions	Soutenir la mise en œuvre, l’exécution et l’évaluation d’interventions en matière de santé de la population dans de multiples lieux	Mettre à l’échelle les interventions efficaces en matière de santé de la population
Financement admissible (par projet)		
150 000 $ à 250 000 $/année	300 000 $ à 750 000 $/année	Jusqu’à 500 000 $/année
Nombre de projets financés		
PSM : 15	PSM : 9	PSM : 4
APS : 37	APS : 11	APS : 7
Portée des interventions financées parmi les personnes ou celles confrontées à des conditions ou des facteurs de risque spécifiques		
PSM : 3 358	PSM : 281 742	PSM : 182 818
APS : 59 081	APS : 21 943	APS : 87 846
Nombre et pourcentage de projets démontrant des changements dans les résultats en matière de santé, les facteurs de protection et les comportements à risque		
N/A*	9 (100 %) projets de PSM	4 (100 %) projets de PSM
8 (22 %) projets d’APS	7 (64 %) projets d’APS^†^	7 (100 %) projets d’APS

*PSM*, volet de financement Outiller les Canadiens – La santé mentale pour la vie; *APS*, volet de financement Atteinte du poids santé dans les collectivités du Canada; *N/A*, non applicable

*Étant donné qu’il s’agissait d’un résultat intermédiaire avec un délai de quatre à six ans et que les formulaires d’évaluation et de rapport du rendement produits par les projets à l’époque ne comprenaient que les données sur la première année de mise en œuvre, de nombreux projets n’étaient pas en mesure de répondre à cette question. Ou les projets n’avaient pas tenté d’améliorer directement les pratiques, les compétences ou les résultats en matière de santé à ce stade, ou cela n’avait pas encore été suffisamment mesuré d’une manière permettant de confirmer de façon irréfutable qu’il y avait eu un changement

†Ce chiffre représente le changement de comportement uniquement pour les projets de la phase 2 financés dans le cadre du volet Atteinte du poids santé dans les collectivités du Canada; 55 % (6/11) des projets ont signalé l’évolution des facteurs de protection parmi les participants. De plus, 45 % (5/11) des projets ont signalé une amélioration du bien-être chez les participants

## Méthodes d’examen et d’évaluation

La SI de l’ASPC a fonctionné pendant environ 11 ans au moyen d’un processus de réflexion analytique et structuré intégré à sa mise en œuvre pour favoriser une culture de l’innovation et de l’apprentissage (Cook & Bradley Dexter, 2021). Les conclusions du présent article sont tirées de ce processus et incluent des faits saillants liés aux quatre éléments du programme. Pour faire ressortir ces faits saillants, les sources de projets et de programmes, y compris des rapports d’expert-conseil indépendant au niveau des projets et des programmes, des produits de connaissance des projets, des rapports de la SI de l’ASPC et des évaluations ministérielles de l’ASPC ont été analysés.

De plus, une surveillance rigoureuse du rendement a permis de saisir les résultats du financement de la SI de l’ASPC liés aux quatre éléments. Les outils et processus de mesure du rendement mis au point pour les examens d’évaluation^14^ des projets financés (Boileau-Falardeau et al., 2021) ainsi que la mesure du rendement au niveau des programmes ont éclairé l’analyse. Bien que des exemples qualitatifs de projets soient fournis pour illustrer les éléments du modèle de financement, le présent article n’inclut pas un aperçu des résultats spécifiques des projets financés.

## Intervention et résultats

Tel que mentionné, la SI de l’ASPC a mis l’accent sur quatre éléments : une approche progressive de financement (subventions et contributions); l’établissement de partenariats; le développement et l’échange de connaissances; et le renforcement des capacités et de l’innovation (ressources, méthodes et outils). Ces éléments ont été orientés au moyen d’une approche de RIDSP reconnaissant l’établissement de partenariats intersectoriels, la multiplicité et la qualité de la mise en œuvre des interventions comme un sous-ensemble de la RIDSP et comme objectif clé, l’échange de connaissances (Riley et al., 2015). Chaque élément a été élaboré, mis en œuvre, surveillé et évalué pour faire avancer les objectifs de la SI de l’ASPC de manière itérative grâce à des travaux complémentaires aux niveaux des projets, des cohortes de projets et du programme. Pour dresser un portrait solide de la SI de l’ASPC, les résultats sont encadrés par les quatre éléments de base (Figure 1 et Tableau 1). Les éléments ont également été influencés par des principes transversaux, notamment l’innovation sociale, l’action multisectorielle et à niveaux multiples, la sécurité culturelle, l’équité en matière de santé et la prise de décision fondée sur des données probantes.

1. Une approche progressive en matière de l’innovation sociale et du changement de systèmes (subventions et contributions)

L’approche de la SI de l’ASPC reconnaît que les interventions novatrices et de haute qualité en matière de santé de la population doivent être élaborées, mises à l’essai et évaluées au cours d’un cycle de développement pluriannuel. Même si l’approche comportait à l’origine deux phases, il a été observé qu’une troisième phase fournirait aux interventions efficaces la possibilité d’être mises à l’échelle pour augmenter leur portée et avoir une incidence sur le changement de systèmes (Bradley Dexter et al., 2021). La phase 1 a soutenu la conception initiale, l’élaboration et la mise à l’essai d’une intervention; la phase 2 était axée sur la mise en œuvre complète de l’intervention dans des lieux supplémentaires, parallèlement à une évaluation rigoureuse; et enfin, la phase 3 a fourni du financement pour mettre à l’échelle l’intervention afin d’en étendre la portée et d’assurer une incidence durable^15^. Une approche de financement à long terme en trois phases (Figure 1) a permis et soutenu les longs processus liés à l’innovation sociale et au changement de systèmes que les modèles de financement traditionnels plus courts ne pouvaient prendre en charge.

Conformément aux recommandations du groupe d’experts sur les subventions et les contributions du Conseil du Trésor, l’approche progressive de la SI de l’ASPC (Encadré 1) était concurrentielle et permettait aux projets de recevoir du financement pendant une période pouvant aller jusqu’à neuf ans, bien que la présentation d’une demande dans le cadre des étapes suivantes se faisait sur une base volontaire et était à la discrétion du projet (Bradley Dexter et al., 2021). Cette approche a facilité la mise en place d’un cadre de financement stable permettant d’établir des partenariats durables et de mettre en œuvre, de mettre à l’essai et d’adapter les travaux tout en effectuant des recherches de grande qualité axées sur la mise à l’échelle et l’incidence à long terme. Les entrevues de fin de projet réalisées avec les projets financés ont révélé que le processus de demande en plusieurs phases a incité l’organisation hôte à tenir compte délibérément de la mise à l’échelle et du changement de systèmes lors de la conception de son réseau de partenariats et de l’élargissement de son intervention.

**Encadré 1:** L’approche progressive de financement de la SI de l’ASPC

• **Phase 1: Le lancement et l’élaboration** se sont concentrés sur la conception initiale, l’élaboration, la mise à l’essai et l’exécution des interventions en matière de santé de la population sur une période initiale de 12 à 18 mois (budgets annuels de 150 000 $ à 250 000 $). La phase 1 a soutenu l’établissement de partenariats acquis et de réseaux entre les secteurs publics, sans but lucratif, communautaire, universitaire et privé. • **Phase 2: L’exécution et l’évaluation** ont soutenu la mise en œuvre complète, l’adaptation et l’évaluation des interventions globales en matière de santé de la population pour de multiples populations, communautés (dans au moins trois administrations différentes) et milieux sur une période allant jusqu’à quatre ans (budgets annuels de 300 000 $ à 750 000 $). La phase 2 a permis la création et le partage de données probantes sur la compatibilité entre les contextes communautaire et culturel, l’état de préparation à la mise en œuvre et l’incidence mesurable des interventions en matière de promotion de la santé de la population. • **Phase 3: Les mises à l’échelle réussies** ont soutenu des efforts visant à augmenter la portée et l’incidence des interventions fructueuses de promotion de la santé de la population évaluées (budgets annuels de 500 000 $ ou moins). Ce financement était disponible pour une période de trois ans afin de renforcer les capacités d’interventions éprouvées, et de créer des possibilités de mise à l’échelle de leur portée pour rejoindre d’autres populations ainsi que de leur incidence sur les politiques et soutenir l’incidence mesurable.	

Au cours de la phase 1, les demandeurs ont été évalués en fonction d’exigences correspondant à une approche axée sur la santé de la population combinée à des indicateurs clés précoces de leur capacité à développer, offrir, évaluer et établir des partenariats. À la fin de la phase 1, les interventions financées avaient rejoint plus de 60 000 individus touchées par des conditions ou des facteurs de risque spécifiques, et certains démontraient déjà avoir produit des changements dans les résultats en matière de santé et les facteurs de risque ou de protection sur une période de 18 mois (Tableau 1). Au cours de la phase 2, les projets ont élaboré des plans d’évaluation et ont élargi leur portée, rejoignant plus de 1 000 communautés dans l’ensemble des provinces et des territoires. Au cours de la phase 3, les demandeurs ont été évalués en fonction de la capacité de leur intervention à être mise à l’échelle. Les critères d’évaluation ont été tirés des recherches et analyses liées à la SI de l’ASPC afin de déterminer des indicateurs de réussite de mise à l’échelle pour les interventions en matière de santé de la population. Ces domaines comprenaient : les données probantes sur l’intervention et l’évaluation, la portée et l’échelle, la capacité organisationnelle, l’établissement de partenariats, l’état de préparation du système, le contexte communautaire, les facteurs de coût, et le développement et l’échange de connaissances (Bradley Dexter et al., 2021).

Comme indiqué, à la fin de la phase 3, 82 % des projets étaient en mesure de poursuivre l’ensemble ou une partie des activités de leur intervention une fois le financement de l’ASPC terminé. De plus, parmi 90 % des projets de la phase 3, il y a eu 44 cas de changement des politiques et pratiques, couvrant un éventail d’administrations locales, régionales, provinciales et territoriales. Grâce à l’approche progressive, les projets ont pu s’adapter, réfléchir et créer des réseaux de partenariats supplémentaires pour avoir une incidence sur les politiques et pratiques tout en augmentant l’échelle vers la durabilité (Bradley Dexter et al., 2021).

(2) Établissements de partenariats

Une solide approche en matière de partenariats a soutenu le financement dans le but de renforcer les capacités de mise en œuvre, de mobilisation des connaissances et d’élaboration de politiques dans plusieurs domaines. Bien que de nombreux bailleurs de fonds soutiennent et surveillent l’établissement de partenariats, la SI de l’ASPC a reconnu qu’il s’agissait d’une composante fondamentale de la mise à l’échelle et du changement éventuel de systèmes. L’établissement de partenariats et la collaboration avec des praticiens, des chercheurs, des décideurs et des organisations communautaires faisaient partie intégrante de la planification des projets et les collaborations réunissaient des administrations locales, des municipalités, des autorités régionales de la santé et des gouvernements provinciaux ou territoriaux, ainsi que des organisations du secteur privé. À la phase 2, près de 40 % des plus de 1 400 partenariats établis dans le cadre des 20 projets financés avaient été maintenus pendant trois ans ou plus. Au fur et à mesure que les projets financés étaient mis à l’échelle au cours de la phase 3, le nombre moyen de partenariats par projet est demeuré stable au-dessus de 50 % et plus de la moitié (58 %) a été maintenue pendant trois ans ou plus. Cet investissement en temps pour établir et entretenir des partenariats était une caractéristique essentielle des projets capables de bien fonctionner au sein des communautés qu’ils desservaient, d’être mis à l’échelle à dessein et enfin, de changer les systèmes. Un extrait d’un récent rapport d’évaluation de la SI de l’ASPC le souligne dans une section « Pleins feux sur un projet » mettant l’accent sur deux projets financés de la phase 2 (Encadré 2).

**Encadré 2:** Projet en vedette: Partenariats

***Outiller les Canadiens – La santé mentale pour la vie*** Le projet *Vers l’épanouissement : améliorer la santé mentale des familles*, dirigé par l’Université du Manitoba, a établi des partenariats avec un large éventail d’intervenants, notamment les ministères provinciaux de la Santé, les régies régionales de la santé, l’Assemblée des chefs du Manitoba et le Groupe consultatif des Premières Nations. Les partenaires ont amené un large éventail d’expérience, de connaissances et de compétences au projet et ont contribué à l’amélioration et la promotion du bien-être mental des parents et de leurs familles. Ces solides partenariats ont en définitive mené à l’exécution de ce projet par l’entremise de la Province du Manitoba pour soutenir la stratégie *Vers l’épanouissement* dans l’ensemble de la province. ***Atteinte du poids santé dans les collectivités du Canada*** *Réseau Quartiers verts / Active Neighbourhoods Canada*, dirigé par le Centre d’écologie urbaine de Montréal / Montreal Urban Ecology Centre, comptait 60 intervenants à la fin de la phase 3 dans les secteurs de la santé, des transports, de la planification, de l’environnement, des services sociaux et de la recherche mobilisés pour mener des activités d’évaluation des besoins communautaires et créer des environnements sociaux et physiques propices au transport actif. (Office of Evaluation, Health Canada and the Public Health Agency of Canada, 2019)	

Une analyse des « partenariats acquis » au sein de la SI de l’ASPC met en évidence les caractéristiques d’une approche solide en matière de partenariats favorisant le changement de systèmes. Les partenariats acquis présentent des partenaires diversifiés, un programme sectoriel public clair, une valeur collaborative démontrée, souvent une mise en commun d’actifs humains et financiers et un engagement envers l’harmonisation et le dialogue, ainsi qu’une approche collective en ce qui a trait au changement de systèmes collaboratifs (Lee & Kavanagh Salmond, 2021). Même si la phase 2 prévoyait l’établissement de partenariats, les premiers apprentissages ont indiqué que plus de temps devait être accordé pour établir des liens significatifs au niveau des projets. Beaucoup de temps et de financement étaient également nécessaires pour réaliser des activités de sensibilisation, rencontrer des partenaires et établir des relations continues, significatives et durables.

Ce changement de cap vers la prise en compte du caractère *acquis* des partenariats a également une incidence sur les efforts de suivi du rendement qui devraient chercher à saisir le mouvement de l’effort de collaboration, y compris l’établissement de l’objectif, plutôt que le nombre de partenaires dans un réseau ou leur portée. Dans ce contexte, la SI de l’ASPC utilise la durée des partenariats comme mesure limitée du caractère acquis, ainsi qu’un commentaire introspectif sur les défis de la collecte et de la contextualisation d’information à des fins de mesure et d’évaluation du rendement (Boileau-Falardeau et al., 2021).

(3) Développement et échange des connaissances

Le développement et l’échange des connaissances (DEC) était orienté de manière à élaborer des stratégies pour partager des produits de connaissances fondés sur des données probantes, des activités et des leçons tirées afin d’avoir une incidence sur l’élaboration des pratiques, programmes et politiques à venir en matière de santé publique. L’accent combiné mis sur les partenariats et l’échange de connaissances a donné lieu à plus de 400 exemples d’application des connaissances générées par les activités des interventions des projets de la phase 2, et plus de 78 % de ces exemples ont directement orienté ou dirigé la mise en œuvre ou l’adaptation d’une pratique, d’une politique ou d’un programme.

Au cours de la phase 3, l’accent a davantage été mis sur l’utilisation efficace des connaissances par les partenaires internes et externes et d’autres intervenants (utilisateurs potentiels des connaissances externes au projet) plutôt que sur la quantité de produits de connaissances. Les projets ont continué de créer et de fournir des milliers de produits et d’activités de connaissances dont un plus grand nombre a été conçu pour avoir une influence sur les politiques, les pratiques et les programmes. Les projets ont cité plus de 200 exemples de la manière dont les connaissances générées par leur intervention ont influencé les politiques, les pratiques ou les programmes. Comme indiqué dans l’encadré 3, les projets de la phase 3 ont pu travailler avec leurs partenaires pour influencer la modification de politiques aux niveaux régional, provincial et territorial et adapter les programmes de santé publique. La SI de l’ASPC a eu une incidence sur divers secteurs, notamment sur des plans de transport municipaux pour encourager l’activité physique, sur des plans régionaux pour soutenir la sécurité alimentaire locale et sur des districts scolaires qui ont adopté un programme d’apprentissage social et émotionnel. Du temps et des ressources ont été consacrés au processus de DEC au niveau des projets puisque la création de plans de DEC était prévue dans le cadre du plan de travail. Le financement à long terme s’étalant jusque sur neuf ans, combiné à l’établissement de partenariats et au DEC, a permis aux projets de repenser leur approche à chaque phase et de continuer à maximiser l’utilisation des connaissances et données probantes dans la prise de décisions relatives aux pratiques et politiques, entraînant des changements par rapport à des problèmes complexes de santé publique.

**Encadré 3:** Projet en vedette: Développement et échange de connaissances

***Outiller les Canadiens – La santé mentale pour la vie*** Les initiatives de développement et d’échange de connaissances du projet *Le quatrième R : Relations saines* dirigé par le Centre de toxicomanie et de santé mentale (CAMH) et l’Université de Western Ontario ont appuyé l’inclusion d’un cours d’études supérieures sur la sécurité dans les écoles pour les étudiants en enseignement de l’Université de Calgary et de l’Université de Lethbridge. Ce projet a également contribué à l’élaboration de politiques et de lois de lutte contre l’intimidation dans les Territoires du Nord-Ouest. ***Atteinte du poids santé dans les collectivités du Canada*** Le projet *Accroître l’incidence et étendre la portée du modèle des centres d’alimentation communautaires* dirigé par les Centres communautaires d’alimentation du Canada a fourni un accès à des connaissances, à de la formation et à des ressources à des organisations communautaires de sécurité alimentaire partout au pays grâce à son programme *Good Food Organizations* (La bonne nourriture n’est qu’un début). Ce programme aide les organisations communautaires de sécurité alimentaire à modifier leurs politiques en matière de programme ou d’organisation. Une évaluation annuelle du programme a permis de relever les exemples suivants de changement de politiques internes dans les organisations membres : • 49 % des bonnes organisations alimentaires ont créé ou approfondi une politique alimentaire saine qui guide les achats et les choix de menu; • 93 % ont élargi leur programme alimentaire pour atteindre plusieurs objectifs dans les domaines de l’accès aux aliments, des aptitudes alimentaires et de la mobilisation communautaire; et • augmentation de 73 % du soutien matériel pour permettre aux personnes à faible revenu et marginalisées de participer à leurs programmes. Le projet *Départ Santé / Healthy Start (DSHS): A multi-level intervention to increase physical activity and healthy eating among young children (ages 3-5) attending early learning programs* dirigé par le Réseau Santé en français de la Saskatchewan (RSFS) a mis en place des équipes de développement et d’échange de connaissances (DEC) pour offrir des séances de formation et d’information aux éducateurs de la petite enfance et aux administrateurs de garderies. Grâce à la promotion des pratiques exemplaires, des champions individuels et des séances de formation d’appoint, plusieurs possibilités de formation et de soutien ont été offertes. À titre de première indication d’utilisation des connaissances, après la formation, 94 % des centres utilisaient le matériel sur l’activité physique et 95 % utilisaient les ressources nutritionnelles au moins deux fois par mois. Le projet *Our Food NL* (Les aliments d’abord) dirigé par Food First Newfoundland a permis la mise en œuvre d’un modèle novateur d’évaluations alimentaires communautaires (EAC). Une EAC aide une communauté à identifier les lacunes et les forces liées à l’accès à une alimentation saine et adaptée sur le plan culturel afin qu’elle puisse lancer des solutions efficaces aux défis locaux en matière de sécurité alimentaire. Cet effort a réuni un large éventail de partenaires aux niveaux communautaire, régional, provincial et national pour relever de graves problèmes en matière de sécurité alimentaire auxquels sont confrontées les communautés. Le modèle d’EAC a eu une incidence durable sur les politiques et les programmes au sein de plusieurs collectivités de Terre-Neuve-et-Labrador. Par exemple, l’EAC a entraîné des changements concrets dans la disponibilité et la diversité des produits frais offerts dans les magasins communautaires. À Rencontre East (accessible uniquement par traversier), un commerçant local a mis en place un système de points selon lequel les clients accumulent des points lorsqu’ils achètent des fruits et légumes frais, et à la fin du mois, le client ayant le plus de points reçoit un prix. En réponse aux conclusions de l’EAC, cette entreprise privée a commencé à offrir des portions individuelles de fruits et légumes pour répondre aux commentaires des personnes âgées (Our Food NL, 2018).	

La mesure de l’utilisation des produits et des activités de connaissance par d’autres intervenants représentait un défi pour les projets; cependant, à la phase 3, tous les projets étaient en mesure de déterminer comment les connaissances générées par leurs travaux influaient sur les politiques et pratiques^16^. Malgré les réussites relatives des projets en matière de DEC, la capacité à influencer le changement de systèmes requiert un public réceptif et adaptable. Le DEC nécessite également de l’expertise, du temps et des ressources pour établir des liens entre le programme de recherche et d’évaluation d’une intervention et les besoins en matière de connaissances d’un concepteur de programme ou d’un décideur. Les futures itérations de ce modèle devraient comporter une plate-forme externe interactive dédiée au DEC permettant aux projets de renforcer leurs capacités afin de soutenir la conception de plans d’évaluation à l’aide d’un plan intégral de DEC, tout en facilitant l’utilisation des connaissances en développant une communauté de praticiens et de décideurs agissant à titre de courtiers et d’utilisateurs des connaissances.

(4) Renforcement des capacités et de l’innovation (ressources, méthodes et outils)

La SI de l’ASPC a soutenu des projets en créant ou en complétant de nombreuses ressources, de nombreuses méthodes et de nombreux outils pour renforcer la capacité et l’accès à l’information par rapport à une variété de sujets, y compris la science de la mise en œuvre, la mobilisation des connaissances, l’incidence collective, le changement des politiques et la mise à l’échelle. Ceux-ci étaient souvent offerts dans le cadre de partenariats et mis à la disposition d’un public plus large (p. ex. webinaires avec le Centre de collaboration nationale des méthodes et outils) ou par le biais de liens officiels entre la SI de l’ASPC et les partenaires des projets (p. ex. présentations techniques de la SI de l’ASPC à l’intention des comités consultatifs des projets). De plus, l’équipe de la SI de l’ASPC a organisé des ateliers annuels de plusieurs jours pour permettre aux projets financés de rencontrer des experts dans divers domaines et d’échanger des leçons apprises structurées sur des sujets liés à la science de la mise en œuvre, à l’établissement de partenariats, à la mise à l’échelle et à l’évaluation. Ces thèmes ont été identifiés par les projets pour répondre à des besoins spécifiques en matière de renforcement des capacités, et à la phase 3, les projets dirigeaient l’élaboration des ateliers annuels, leur animation et la création de leur contenu.

Une lacune en matière de capacité d’évaluation a été décelée au cours de la phase 1 et la majorité des projets se sont associés à une université ou à un chercheur pour apporter la rigueur nécessaire à l’évaluation de leur intervention et recueillir suffisamment de données aux fins de la RIDSP. De plus, la SI de l’ASPC a appuyé l’élaboration de plusieurs ressources d’évaluation et a fait appel à un expert en évaluation pour fournir du soutien aux projets par rapport à l’analyse du retour sur investissement et l’évaluation de l’influence des politiques ainsi que du soutien individuel par rapport à des plans d’évaluation personnalisés. Les contextes des projets, les populations desservies par ceux-ci et leurs approches d’intervention étaient très diversifiés. C’est pourquoi une boîte à outils ou une ressource générique pour la RIDSP n’était pas aussi efficace que des approches individuelles. Dans ses futures itérations, le renforcement des capacités et des soutiens personnalisés sera intégré au modèle de financement, dès la phase 1, puis intégré et soutenu davantage au moyen d’un centre externe de développement et d’échange de connaissances. Il était également difficile de dégager l’incidence des outils de renforcement des capacités, et les futures itérations du modèle de financement relieront cette composante au DEC et au travail de partenariat en faveur du changement de systèmes.

## Répercussions

Le modèle de la SI de l’ASPC a orienté la conception d’autres initiatives de financement de l’Institut de la santé publique et des populations des Instituts de recherche en santé du Canada (CIHR, 2016a; CIHR-IPPH, 2010) et l’initiative *Voies de l’équité en santé pour les Autochtones* (CIHR, 2016b) ainsi que les programmes de financement subséquents de l’ASPC *Contribuer à la santé des survivants de violence familiale*, le *Fonds pour la santé mentale des communautés noires* et le *Fonds d’investissement communautaire sur la démence* (Office of Evaluation, Health Canada and the Public Health Agency of Canada, 2019).

Un certain nombre de leçons importantes apprises au cours du parcours de la SI de l’ASPC sont utiles à ceux qui administrent et gèrent des programmes de financement stratégique pour la promotion de la santé au niveau des individus et des systèmes. Premièrement, l’établissement de solides partenariats acquis a été essentiel dans l’exécution, la mise en œuvre, la mise à l’échelle et la pérennité des projets dans les deux volets de financement. Ces partenariats ont mené à un échange bilatéral de précieuses connaissances qui ont permis d’améliorer les pratiques en vue de modifier les systèmes. Les analyses des partenariats acquis ont démontré la nécessité de saisir le mouvement collectif et le programme commun (Lee & Kavanagh Salmond, 2021). Les entretiens de fin de projet des projets de la phase 3 ont également souligné l’avantage de disposer de temps et d’espace pour établir des partenariats afin de concentrer les efforts en amont sur le changement de systèmes. La souplesse et le soutien en matière de partenariat et, par extension, de développement de relations et d’activités d’établissement d’un programme partagé pourraient favoriser la mise à l’échelle et la durabilité des programmes.

Deuxièmement, l’intégration d’expertise en évaluation à chaque projet financé a permis de s’assurer que les interventions sont mises à l’essai pour mieux comprendre l’incidence de leurs activités, enrichissant les connaissances sur les approches efficaces. La SI de l’ASPC a fourni du soutien pour mieux comprendre ce que l’on entendait par « mise à l’échelle » (Bradley Dexter et al., 2021) et comment évoluer vers la modification des politiques et des systèmes. Les projets ont donc pu prendre les mesures nécessaires pour élargir leurs interventions et accroître leur pérennité.

Troisièmement, l’approche progressive de la SI de l’ASPC intégrait la souplesse nécessaire pour permettre au programme et aux projets de cerner ce qui avait fonctionné et ce qui n’avait pas fonctionné à la fin de chaque phase et d’en tirer des leçons. Des correctifs ont donc pu être apportés en cours de route afin d’améliorer la réussite globale.

Enfin, la durée du financement et la possibilité de financement prolongé au cours des trois phases ont été considérées comme particulièrement essentielles pour ce type de programme puisque les projets tentaient de créer des changements durables dans le système, ce qui nécessite des années d’engagements en matière d’investissements (Office of Evaluation, Health Canada and the Public Health Agency of Canada, 2019).

Des défis ont également été révélés tout au long de ce processus itératif. Bien que la SI de l’ASPC ait appuyé des projets visant à remédier à des inégalités en matière de santé, il était parfois difficile d’en saisir l’incidence de manière significative, en partie en raison de la complexité et de la diversité du contexte au niveau des projets. Les versions futures de ce programme de financement incluront des possibilités et méthodes au début des cycles de financement pour saisir des données sur la façon dont le projet aborde les inégalités en santé et sur son incidence sur ces dernières.

De plus, malgré la réussite relative de l’intégration du DEC au niveau du programme à des fins d’apprentissage continu, la profondeur et la portée des travaux ont posé des défis. Les versions futures du modèle de la SI de l’ASPC étudient la possibilité de ressources supplémentaires pour soutenir une organisation externe qui renforcera le soutien aux projets afin d’élaborer de rigoureux plans d’évaluation, et de développer des expériences de partenariat de collaboration, des apprentissages partagés et un échange de connaissances entre les projets financés et en dehors de ceux-ci pour favoriser un environnement plus propice à l’innovation sociale en vue de modifier les systèmes.

## Conclusion

Les programmes de subventions stratégiques et adaptables pourraient soutenir des solutions novatrices et locales pour s’attaquer à des problèmes complexes de santé publique. Le modèle de la SI de l’ASPC a appuyé l’action locale créative afin de favoriser des changements positifs et durables dans les systèmes et au sein des administrations des communautés du Canada et d’ailleurs. Le modèle de la SI de l’ASPC y est parvenu en utilisant une approche progressive mettant l’accent sur l’établissement de partenariats, le développement et l’échange de connaissances et le renforcement des capacités et de l’innovation. Pour les quatre éléments de base du modèle de la SI de l’ASPC, les données probantes provenant de solutions novatrices locales à des défis complexes ont influencé et orienté les politiques et pratiques dans divers secteurs et l’ensemble des administrations. Les réflexions tirées de la SI de l’ASPC et les résultats du financement sont présentés dans le présent document pour encourager davantage de dialogue sur l’octroi de subventions et les programmes en matière de promotion de la santé entre différents paliers de gouvernement et des organisations communautaires qui cherchent à réduire les facteurs de risque, à promouvoir les facteurs de protection et à influer sur leurs déterminants sous-jacents de la santé au moyen d’interventions en santé de la population.

Les réalisations produites grâce au soutien financier échelonné à plus long terme au niveau des programmes et des projets en ont soutenu la conception, la mise en œuvre et l’éventuelle mise à l’échelle, et ont favorisé l’établissement de partenariats solides et acquis essentiels à l’échange de connaissances afin d’améliorer les pratiques et de soutenir les politiques de promotion de la santé. La SI de l’ASPC a combiné l’art de l’innovation et de l’amélioration continue et la science de la bonne mise en œuvre en matière de santé publique pour former une base intentionnelle afin d’orienter la conception stratégique d’un modèle de financement permettant de relever les défis complexes de santé publique auxquels sont confrontées les communautés du Canada.

### Acknowledgements

The authors would like to thank the PHAC-IS-funded projects for their engagement with the national program and the impact they have had with individuals, families, and communities across Canada. We are thankful for the time projects dedicated to complete annual reporting requirements that have informed the development of this article and made a considerable contribution to the PHAC-IS funding model. We would also like to acknowledge the PHAC-IS team, both past and present, for their review and insights with regard to this article. Special thanks to Christine Czoli and Elizaveta Oulman for their work on the status of each funding stream and the valuable insight that this provided. Thank you to Andrea Simpson and Vicky Laramée for their reviews, edits, and translation work for this article and the PHAC-IS Special Issue. We would also like to recognize the contributions of Susan Stevenson, Doug Crossman, and Brian Bell, who were early innovators in the design and implementation of the PHAC-IS. Finally, we would like to thank PHAC and the Mental Health and Wellbeing Division within the Centre for Health Promotion for supporting the dissemination of learnings and reflections on the PHAC-IS.

### Remerciements

Les auteurs tiennent à remercier les projets financés par la SI de l’ASPC pour leur participation au programme national et l’incidence qu’ils ont eue auprès d’individus, de familles et de communautés partout au Canada. Nous sommes reconnaissantes pour le temps consacré par les projets à la production des rapports annuels exigés qui ont guidé la rédaction du présent article et apporté une contribution considérable au modèle de financement de la SI de l’ASPC. Nous tenons également à remercier l’équipe de la SI de l’ASPC, passée et présente, pour avoir examiné le présent article et formulé des commentaires. Un merci spécial à Christine Czoli et Elizaveta Oulman pour leur travail sur l’état de chaque volet de financement et les précieuses données fournies. Merci à Andrea Simpson et Vicky Laramée pour leur soutien dans les révisions, modifications et traductions pour cet article et le numéro spécial SI de l’ASPC. Nous tenons également à souligner les contributions de Susan Stevenson, Doug Crossman et Brian Bell, qui ont été parmi les premiers innovateurs dans la conception et la mise en œuvre de la SI de l’ASPC. Enfin, nous tenons à remercier l’ASPC et la Division de la santé mentale et du bien-être du Centre de promotion de la santé pour avoir soutenu la diffusion des apprentissages et réflexions liées à la SI de l’ASPC.

## Data Availability

All data, reports, and materials were made available from the PHAC-IS funding program and project-level-completed reports.

## Footnotes

1 PHIR refers to the use of scientific methods to produce knowledge about policy and program interventions that operate within or outside the health sector and have the potential to impact health at the population level. This intervention research approach focuses on building knowledge about how the intervention process results in change and the context in which the intervention works best and for which populations.

2 The PHAC-IS was provided with an annual average of approximately $6.2 million which was used to fund projects that generated knowledge and evidence to improve health and health equity at the population level.

3 This work is ongoing under the Mental Health Promotion Innovation Fund, launched in 2019.

4 A project that was successful through the 3 phases would have received an average of $3.5 million over the 9 years of funding. Please see Table 1 for eligible funding totals per project during each phase.

5 Examples from funded projects are drawn upon to illustrate components of the PHAC-IS model, but this article does not include specific outcomes or evaluation results of the funded projects.

6 Each of the funded projects completed the following documentation and various data sources were used to compile these findings, including: (1) the PHAC-IS’s annual reporting tool (ISART) (formerly called the Performance Measurement and Evaluation Reporting Tool [PERT]) which was completed by projects annually to monitor project activities and to assess the impact of interventions on the health of participants and their communities; (2) the final report template (which was submitted in lieu of the ISART in the final year of each phase); (3) evaluation research reports produced by funded projects; and (4) end of funding interviews carried out 3 to 6 months after funding ended.

7 Details and funding amounts included in Text Box 1.

8 Projects were supplied the tool developed by Knott and Wildavsky’s Stages of Knowledge Translation (Knott & Wildavsky, 1980) and reported on KDE impact within the annual reporting tools.

9 La RIDSP est l’utilisation de méthodes scientifiques pour produire des connaissances sur les interventions, sous forme de politiques et de programmes, qui existent dans le secteur de la santé ou à l’extérieur de celui-ci et qui pourraient avoir une incidence sur la santé de la population. Cette approche de recherche interventionnelle met l’accent sur le renforcement des connaissances quant à la façon dont le processus d’intervention génère des changements et au contexte qui a le mieux fonctionné pour l’intervention et pour certaines populations.

10 La SI de l’ASPC a reçu une moyenne annuelle d’environ 6,2 millions de dollars qui ont été utilisés pour financer des projets générant des connaissances et des données probantes qui améliorent la santé et l’équité en santé au sein de la population.

11 Ces travaux sont en cours dans le cadre du Fonds d’innovation pour la promotion de la santé mentale, lancé en 2019.

12 Un projet ayant terminé les trois phases aurait reçu en moyenne 3,5 millions de dollars au cours des neuf années de financement. Veuillez consulter le tableau 1 qui indique les totaux de financement admissible par projet au cours de chaque phase.

13 Des exemples de projets financés sont utilisés pour illustrer les éléments du modèle de la SI de l’ASPC, mais le présent article n’inclut pas de résultats ou de résultats d’évaluation spécifiques des projets financés.

14 Chacun des projets financés a produit les documents suivants et diverses sources de données ont été utilisées pour compiler ces constatations, notamment : 1) l’outil de rapport annuel de la SI de l’ASPC (anciennement appelé outil de rapport d’évaluation et de mesure du rendement [OREMR]) utilisé annuellement par les projets pour assurer le suivi des activités de projet et évaluer l’incidence des interventions sur la santé des participants et de leurs communautés; 2) le modèle de rapport final (soumis à la place de l’outil de rapport annuel de la SI de l’ASPC au cours de la dernière année de chaque phase); 3) les rapports de recherche d’évaluation produits par les projets financés; et 4) les entretiens de fin de financement réalisés trois à six mois après la fin du financement.

15 Détails et montants de financement indiqués dans l’encadré 1.

16 L’outil développé par Knott et Wildavsky sur les étapes de l’application des connaissances (Knott & Wildavsky, 1980) a été fourni aux projets qui ont rendu compte de l’incidence du DEC au moyen des outils de production de rapport annuel.

## Code availability

N/A.
